# An Inspection of the Life Cycle of Sustainable Construction Projects: Towards a Biomimicry-Based Road Map Integrating Circular Economy

**DOI:** 10.3390/biomimetics6040067

**Published:** 2021-11-29

**Authors:** Kimberly Beermann, Miguel Chen Austin

**Affiliations:** 1 Universidad Tecnológica de Panamá, Panama City 0801, Panama; kimberly.beermann@utp.ac.pa; 2 International Association for Hydro-Environment Engineering and Research (IAHR), Panama Young Professionals Network (YPN), Panama City 0801, Panama; 3 Faculty of Mechanical Engineering, Universidad Tecnológica de Panamá, Panama City 0801, Panama; 4 Centro de Estudios Multidisciplinarios en Ciencias, Ingeniería y Tecnología (CEMCIT-AIP), Panama City 0801, Panama; 5 Sistema Nacional de Investigación (SNI), Panama City 0801, Panama

**Keywords:** sustainable construction, project management, sustainability, circular economy, biomimicry, road map, life cycle phases

## Abstract

According to the National Energy Plan in Panama, the construction sector is one of the most prosperous and impactful sectors in the economy and it is expected to expand due to population growth by almost 300% by 2050. However, this sector must work on the transition towards sustainability and resilience in the face of climate change, since its growth implies a high consumption of resources and the contribution of greenhouse gases. The need to establish practices and strategies that embrace the dimension of sustainability and a circular economy is imminent. Currently, there is little guidance in the reference framework beyond certifications in planning, management and evaluation tools for its implementation. Different studies vary in the number of phases and considerations for projects. Therefore, the present work proposes the development of a unified road map, with defined phases, practices and indicators based on principles inspired by nature, such as biomimicry (Greek words: “bio” means life and “mimesis”, imitation), and focuses on a circular economy, validated by construction professionals, where strengths, opportunities, skills and threats are identified with a high level of acceptance. This contributes to strengthening the field of sustainable construction project management and a precedent for Panama.

## 1. Introduction

The construction industry has a great economic influence and presents great opportunities, unlike other sectors, to face the challenges of climate change [1,2] and global challenges. For this reason, it is essential to adopt practices based on sustainability and circular economy principles at all stages of the process, since it involves high consumption of resources and negative impacts on the environment [3]. Studies indicate that the construction industry is responsible for about 50% of carbon dioxide emissions into the atmosphere; further, 20–50% of its natural resource consumption and 50% of its solid waste generation cause environmental impacts [4]. It is expected to expand by 50% in global terms, due to population growth and the demand for buildings and energy [5]. In the case of Panama, this population increase will be almost 300% by 2050, according to the National Energy Plan [6], which would have a proportional impact on resource consumption and greenhouse gas emissions. Thus, it is imminent that the construction industry must act with commitment and responsibility, given its contributions to the environment, society and the economy.

There are various definitions of “sustainable construction”, but it is positioned as a relevant contemporary issue and aligned with the efforts needed to achieve sustainability and development [7]. Sometimes its focus is limited to environmental dimensionality, but it must encompass all three pillars: environmental, social and economic aspects. The Construction Research Institute (BRE) specifies the approaches under the three pillars of sustainability [8]:

- Environment: reduction in negative impacts on the environment through the selection of renewable materials, management and minimization of waste and adoption of practices for improvement and environmental protection;
- Economy: increased efficiency and growth through the efficient use of resources (materials, energy, water, etc.);
- Social: meeting the needs of the population and social groups involved in the construction process, guaranteeing the satisfaction of all interested parties, including the inhabitants of the project’s area of influence.

This integration of sustainability must occur in all the processes of the life cycle of construction projects and their management, such as initiation, execution, monitoring, control and shutdown [9]. The link of project management with the sustainable environment is an opportunity to explore and identify components, structure and defined integration processes. As found in [10], Silvius, G. supports the management of sustainable projects such as “the management of change-oriented to the project in policies, assets or organizations, taking into account the economic, social and environmental impact of the project, its result and its effect, for the present and future generations”. Therefore, determining the management factors and processes in the framework of sustainable construction, in addition to having multiple benefits in terms of achieving prosperity without compromising the lives and resources of future generations, will respond to raising awareness of the feasibility of application and notion of both costs and risks [9,11].

The reasons for not investing in the change from traditional to sustainable construction is the complexity of design and increased costs [12], but, according to studies, this can be considered an additional success factor in terms of scope, time and costs [13]. In addition to the results already proven in operation, such as reducing 50% of energy consumption in green buildings, these tend to be more durable and have fewer maintenance requirements. The review includes a study that illustrates that an investment of 2% in sustainable construction can produce long-term savings of more than ten times the amount of investment [11]. Due to this profitability of implementation, it is important to contribute to the orientation in the construction process, essentially, in the initial phases (feasibility and design), to intervene less in the operation phase and avoid having to carrying out repairs, which causes low costs [2,14]. Similarly, we take into account the perspectives of administration, planning and the project and product life cycle [2,15].

The findings in the reviewed literature are varied, whereby the relevance of the topic has been recognized but different directions have been developed; some authors focused on the qualities of planning as well as aspects of control and leadership related to the “triple result” [14,15], while others examined preliminary proposals for decision-makers and outlined processes with variation in the quantity of the phases to be carried out and their factors or indicators. In addition, the latent presence of qualification systems for green buildings (LEED and BREAM, among others) and ISO international quality standards related to the “triple result” has been considered [13]. However, for the latter, emphasis has been placed on the inclination for the environmental dimension over the social and economical ones, its sustainable scope being questioned [2,16].

Considering previous studies and the diversity of frameworks and taxonomies found to integrate sustainability in construction and project management, the objective is to define a road map that includes the triple bottom line and extends through the concept of circular economy, which works with biological processes that emulate nature, for the transition from a linear to a circular economy [17,18]. In the articles here reviewed, the circular economy is considered to be a tool to be adopted in order to perform a successful transition to sustainable construction [19], since it is a restorative or regenerative industrial system by intention and design [20], thus complementing the triple factor balance or result approach.

Similarly, the biomimetic approach is included to solve challenges with innovative approaches that aim at making constructions sustainable [21]. This is based on the exploration of nature as a mentor, measure and model that influences both the theoretical or conceptual design-related decisions in any project and the way to approach the built and natural environment through design, materials and technologies [1,22].

The relevance of the topic is currently recognized, but the approaches researchers have employed over the years are diverse and the scope of sustainability is also debatable. Studies have been developed in different directions, such as the quality of planning, process schematization and international standards, as well as green building rating systems (LEED and BREAM, among others). That is why this research study aims to propose and evaluate a road map with a definition of phases, practices and indicators in terms of the triple bottom line (profit, people and planet), circular economy and biomimicry, to unify definitions, considerations and challenges for sustainable construction taking inspiration from nature for problem solving.

This document is divided into three parts. It starts by presenting a review of different frameworks regarding the methodologies used to design sustainable construction projects. This is followed by a presentation of the biomimicry methodology, linked to the circular economy concept, to address the challenge of unifying the diversity in the approaches relative to sustainable construction projects and process definition. Here, a road map based on a named “Biocircular Model” is proposed, with the biomimicry-based strategies’ influence on the sustainable construction process and definition being highlighted. Finally, the “Biocircular Model” is applied to each phase in the construction project and evaluated by experts in the local field via a survey together with a SWOT analysis to support the proposed model and road map.

## 2. Inspection of the Methodologies Used for the Design of Sustainable Construction Projects

The inclusion of sustainability in construction is a topic of interest and great opportunities for the future in the face of global environmental challenges, but directions of its processes, indicators and factors have not been formally defined within the project management framework. For this reason, in order to define the road map, it is necessary to know the barriers and opportunities already identified in the field. How does the literary review define sustainability in construction? Is the process of its inclusion by stages analyzed? Are there metrics for its evaluation? These are the main questions that guided our the literary review, with the ojective of establishing the transition to sustainable construction, complemented with the biomimetic approach and circular economy.

### 2.1. Literature Search Strategy

**Figure 1:**
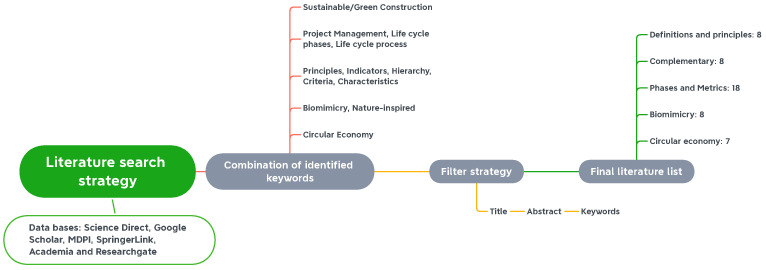
Literature search strategy.

Different databases were used to carry out the search on sustainable construction and link it with biomimicry and the circular economy, such as ScienceDirect, Google Scholar, SpringerLink, Academia and Researchgate, applying Boolean operators and the combination of keywords (see Figure 1). The main approaches were “sustainable or green construction”, “project management or life cycle phases”, “principles, indicators, criteria”, “Biomimicry” and “Circular Economy”. The filtration was first given by the title, then by the abstract and, finally, by the full text. For this study, around 49 documents were considered, of which those that only treated context and principles were useful for the state of the art and domain of the subject. The phases and metrics, biomimicry and circular economy were the most influential concepts for this project.

In this way, we began by understanding how sustainability is defined in construction, analyzing a selection of 20 articles with phases and metrics (Table 1). There was no more than 80% agreement among the elements identified, which illustrates the variation in the orientation of sustainable construction. Occurrence from 55% to 75% was found for the following: the minimization of construction’s impact on the environment [2], decision making considering green factors in all phases of a project [23] and the three pillars or “triple result” of sustainability—environment, economy and society. These are determining factors for construction activities to achieve sustainable development and minimize environmental degradation.

With a 50% frequency, we found resource efficiency and a new process approach [31], energy efficiency and consumption, and waste generation. Health and safety were found in the minimum range, which allows us to reflect on compliance with the dimensionality of sustainability with these mainly social factors.

### 2.2. Comparative Analysis of the Phases and Metrics Considered in Sustainable Construction Projects

**Figure 2:**
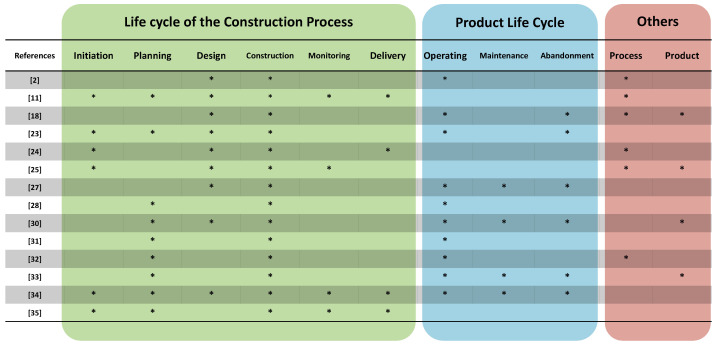
Comparison of phase inclusion for the process and product’s life cycle process.

In the literary review, few documents focused on the stages of the process. Therefore, it was considered necessary to analyze the phases involved, their quantity and definitions (Figure 2) in those studies which did so in the form of a proposal and to find patterns. In the 14 papers selected, there were only mentions of the phases involved, since the authors’ focus was on factors or indicators.

The predominantly mentioned phase was duly execution (construction), while planning and design were viewed jointly. The monitoring and control phases, as well as the delivery phase, were mentioned less frequently. However, in [36], the necessary approach for the road map is linked to the management of imminent projects and the phases that monitor the performance and fulfillment of goals must be included.

Additionally, the vast majority of documents focused on buildings exclusively. This allowed them to explore a product’s life cycle and its stages, such as operation and maintenance. Among the instruments used formally, there are certifications such as the green building qualification systems (GBRT), used to evaluate the sustainability of buildings and ISO management standards [5].

The need to integrate sustainability in the construction sector is clearly defined, but there are no defined reference frameworks for its implementation in project management, life cycle and objectives beyond certifications for evaluation.

For this reason, most of the articles make proposals and contemplate the triple factor or triple bottom line. These articles include a decision matrix with hierarchical analysis [23], proposals for quantitative and qualitative indicators with applicability to case studies [7], definitions of a phase approach together with the green supply chain [25] and the establishment of indicators by construction factors [28].

### 2.3. Applicability of the Biomimetic Approach and Circular Economy in Construction Projects

Biomimicry is the study of and inspiration from biological components and natural processes to solve problems, understanding how they survive, function and evolve in a self-sustaining way [12,21]. Serving as a “model, mentor and measure”, biomimicry has three levels, namely, organism, behavior and ecosystem, and it can be applied in forms, processes and systems [1]. For a long time, biomicmicry has been employed trying to solve problems of food and shelter, as well as others; as a result, we have obtained models, patterns and solutions that have been tested and have successfully, creatively and sustainably solved challenges for humanity over time [37,38].

The motivation for the application of biomimicry in research has had a great increase in recent years, with the aim to find those innovative solutions hidden in nature—mainly in the architecture and engineering fields, whose goal is to employ sustainability for human development [38,39]. Two approaches are presented in biomimicry to solve problems. The “problem-based approach”, which involves solving the design problem by identifying the principle with which another organism or ecosystem solves it. The second approach, i.e., the “approach based on the solution”, consists in solving the problem through the identification of a particular behavior, function, or characteristic in an organism or ecosystem [12,21]. For this research study, the “design looking at biology” or “problem-based approach” is addressed and the “BioGen” design methodology [40] is applied, since this methodology has improved the investigation of the strategies of nature and the extraction of fundamental principles to establish design concepts, as well as the integration of strategies of different organisms to obtain enhanced solutions [40].

## 3. Materials and Methods

**Figure 3:**
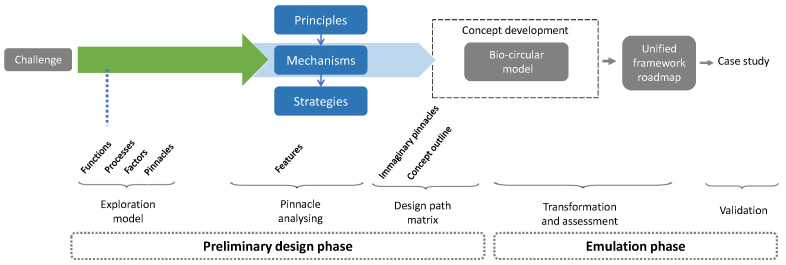
Schematic of the methodology implemented.

The methodology (see Figure 3) is divided into two parts. The preliminary design phase consists of conducting an exploration and investigation of organisms and ecosystems based on challenges, similar functions, extraction and abstraction of principles through the exploratory model, the pinnacle analysis matrix and the design path matrix. We look at the biological domain, where the analysis of solutions to deal with current problems and challenges in sustainable construction are managed and analyzed [41]. Then, the emulation phase is where the ideas are transformed into designs, constituting the biocircular model, where the resulting functions are identified and classified at both the target and application levels and then validated for decision making [40,41]. This validation is given through a survey distributed to 27 construction professionals with different positions and years of experience.

### 3.1. Conceptualization of a Frame of Reference with a View to Sustainability

To apply the methodology, the problem of diversity in the approaches to sustainable construction and the definition of its processes are presented. Therefore, “the three pillars of sustainability” or “triple result” are presented as challenges; environment, economy and society are oriented, in order, to protection, harmonization and well-being, to ensure that construction has a sustainable scope.

#### 3.1.1. Biomimetic and Circular Economy Approach

**Figure 4:**
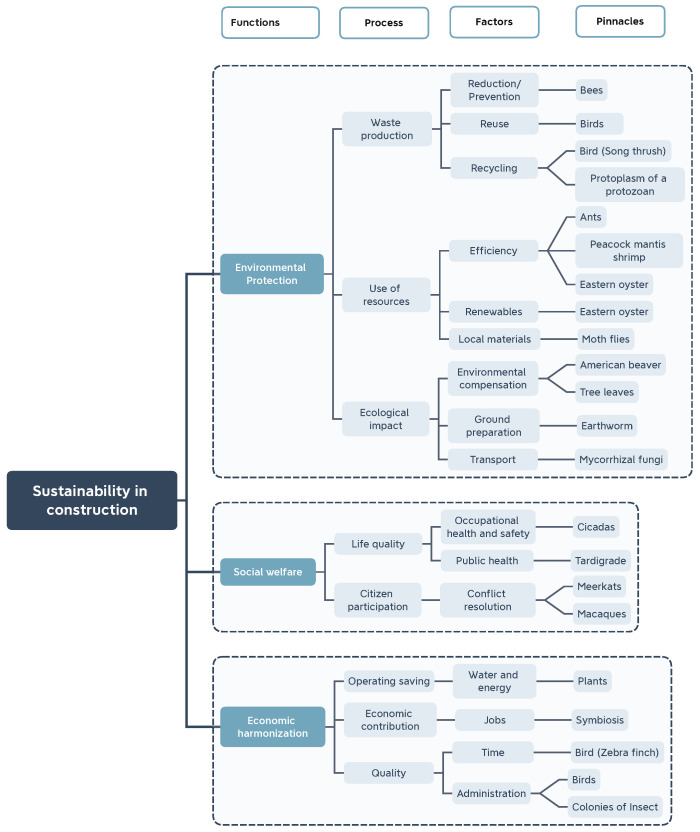
Exploration model connecting challenges and pinnacles.

The first stage within this phase is the exploratory model (Figure 4), which establishes four hierarchical levels for each challenge, establishing the functions in the first level, the relevant processes in the second, the influencing factors in the third and the biological entities (called pinnacles) are presented in the fourth. These represent an example of a specific function, process, or factor. The AskNature platform of the Institute of Biomimicry was used to find nature’s solutions to the challenges.

The first challenge is the protection of the environment, where fundamental processes were identified. Firstly, the production of waste and the hierarchy of sustainable waste management was taken into account [30], with the following factors being prioritized: reduction in/prevention of the generation of waste; reuse of materials and recycling of materials; reduction in consumption of less natural and material resources; reuse of materials that are in good condition and fulfill their original function; and recycling to process materials to obtain the same or lower quality necessary to achieve the task [18]. For this process, searches were carried out with keywords such as “waste” and “build” and with the exploration of the category of innovations in construction; this returned more than 60 results in the categories of optimization of shape/materials and physical assembling, obtaining 12 preselected pinnacles, such as birds, hornets and bees. The use of resources, according to the efficiency and duration, was the second process considered, with the quality of “renewable” considered as a factor, taking into account ecological components and referring to the opportunities to have environmentally friendly materials; in the context of materials sourced from local regions, we considered the reduction in emissions from transport and the support of the local. For the search, logical connections were made with “materials”, “resources” and “construction”, obtaining 41 results for review; among these, a preselection of 8 pinnacles was made for analysis, among which we highlight the eastern oyster, ants and birds.

The last process considered was ecological impact, which involves the existence of environmental compensation for the pollution generated and a correct preparation of the land and transport, which is obtained efficiently with low carbon emissions. In this, reviews of “solutions for climate change” and searches with logical connections such as “build and maintain the community” and “life-friendly transport” were carried out, with 18 results and 6 elements preselected for analysis, such as beavers, worksheets, trees, termites and worms.

These processes and factors have a great environmental focus, but working to protect the environment also affects social well-being; further, finding more efficient materials, reducing them and providing support with the selection of local sources support the economy.

In social welfare, the first process considered was quality of life, encompassing occupational safety and hygiene, aligned with the protection of employees, training and occupational safety; public health, focused on the impact of inhabitants within the project’s area of influence, opportunities for improvement and well-being, was also considered. As another process, the participation of the citizenry is itself part of construction and its orientation is aligned with the resolution of conflicts among interested parties [32,34]. Regarding the search for pinnacles, logical connections of words such as “build and protect from physical damage” and “build and maintain the community” were made with the focus of coordinating, resulting in 26 solutions and 4 preselected elements for analysis, such as cicadas, meerkats, macaques and tardigrades.

Finally, economic harmonization, with processes such as operational and water and energy saving processes, is part of the economic benefits of being sustainable. In addition, economic contribution implies the generation of jobs and that, in the same way, has to do with the social sphere and people’s quality of life. Finally, we considered quality, in terms of administration, its performance and capacity [34]. To identify pinnacles, word connections, such as “efficient design,” “fast build”, “quality of construction” and “symbiosis” (the latter being an agreement to obtain benefits, applied to the concept of receiving income for work performed), were made. There were 86 results and quick filters were applied, with short descriptions of the strategies. As a preselection for analysis, there were six pinnacles.

The analysis of the preselected pinnacles continued through the identification of the strategy and its mechanism, extracting its main principle and the characteristic by which it is carried out [40].

**Figure 5:**
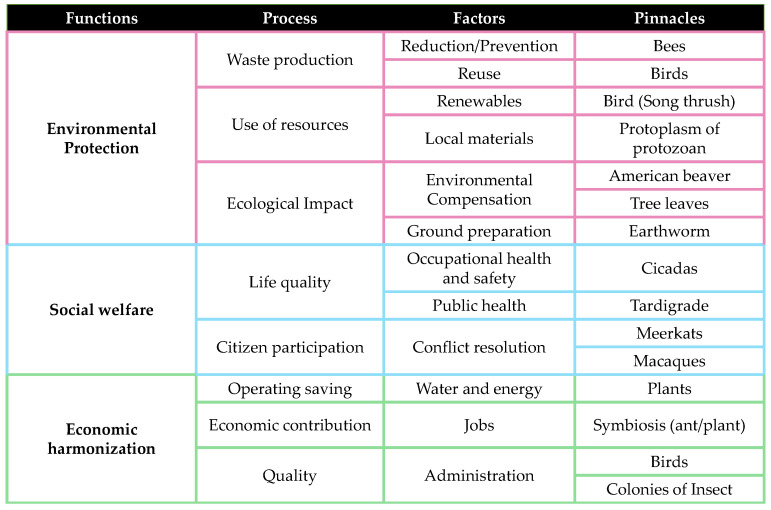
Selected pinnacles from the exploratory model.

For the selection of the fundamental pinnacles (Figure 5), the processes identified for the three challenges were maintained. Among the factors, priority was given, in the case of waste production, to the main factors in the hierarchy of integral waste management [30], highlighting reduction and reuse. For the ecological impact, the transport factor was discarded within the first challenge, since the choice of local materials applies its concept. In the case of the quality process for economic harmonization, the time process was discarded, since it is binding on management and the pinnacle had already been analyzed.

The number of pinnacles required depends on the challenges set and the corresponding solutions provided by the pinnacles, which may be entirely novel for generalities [40].

The strategies of the selected exploration model routes are covered by challenges and processes and, for each, the selected pinnacles are mentioned:

(a) Environmental Protection

- Waste production: birds and bees were chosen. Birds handle construction as a complex process due to their experience and observation, which includes the efficient use of the materials at their disposal and the use of decomposed wood for their nests. Bees forge their hives with the principle of storing the greatest amount of honey with the least building material (wax).
- Resource Utilization: The sacworm was selected for using environmental materials such as twigs, leaves and silk to construct boxes with spiral patterns for protection. On the other hand, the oriental oyster was selected for its creation of a kind of cement from calcium carbonate with softer and stickier proteins; they withstand strong tides and manage to hold their colonies together.
- Ecological impact: beavers, tree leaves and earthworms were chosen. Beavers were chosen for being ecosystem engineers, managing to model entire landscapes including habitats and damaged streams by constructing their dams. Tree leaves can absorb organic compounds from the atmosphere and break them down to be less harmful. Lastly, earthworms are decomposers that add air and disperse nutrients in the soil as they dig; these consume dead organic material, such as leaves and roots and, after consuming it, they break it down and excrete it in the form of nutrients.

(b) Social welfare

- Quality of life: For occupational safety and hygiene, we chose the pinnacle of cicadas, since they expel dirt and water through nanoscale protrusions surrounded by air pockets that attract water droplets. In public health, the tardigrade was selected for its characteristics of protection from extreme environmental conditions through cryptobiosis (i.e., quarantine).
- Conflict solving: Meerkats were selected because of how they manage conflicts—by taking turns in leadership—and macaques because they use a simple, clear and inclusive voting process to stay together as a group.

(c) Economic harmonization

- Operational savings: Plants were chosen as the pinnacle, since their antenna of light capture allows it to be efficient from a quantum point of view, thanks to the high density of pigments and the design of long states of excitation. An addition reason for this selection is the ability, in Bromeliaceae, to capture water and nutrients in a storage tank through hydrophobic leaf surfaces.
- Economic contribution: The symbiosis was selected and the plant/ants agreement was taken as an example; plants provide shelter and other services, while the ant provides nutrients.
- Quality: For the management context, birds were selected for handling and identifying the construction process as complex. Speed and construction materials are also important to them, as they influence their ability to reproduce.

With the pinnacles already selected, we proceeded with the pinnacle analysis matrix and with the design path matrix stages. The design matrix seeks to evaluate an imaginary pinnacle for each category, which are: process (identified for the exploratory model and selection), flow (if they do it actively or passively), adaptation of the process, the scale, the environmental context, that is, the climate, the morphological characteristics and, finally, the circular economy. Five actions of the ReSOLVE framework [17] were placed as characteristics, where:

- “Regenerate” refers to the change to renewable materials and energies, the restoration of ecosystem health and the return of biologically recovered resources to the biosphere.
- “Sharing” is about sharing assets, driving reuse or second hand and prolonging the life of products through maintenance, durable design and upgrades.
- “Optimize” consists of increasing the performance or efficiency of a product, removing waste in the production and supply chain, and alludes to optimization.
- “Cycle” refers to remanufactured products or components, digested anaerobically, recycled materials and biochemicals extracted from organic waste.
- “Exchange” refers to replacing old materials with advanced non-renewable materials, applying new technologies and choosing new products or services.

It should be noted that the action of virtualizing was not considered in the ReSOLVE framework due to its limited applicability for pinnacle analysis, but, together with the elements obtained from the design path matrix, support technologies can be identified.

The “X” symbols are placed when a characteristic is applied to each pinnacle in order to identify the imaginary pinnacle that has the most dominant characteristic by category. This procedure is utilized to reduce the complexity of the solutions found, where the imaginary pinnacle acquires its functions [40].

**Figure 6:**
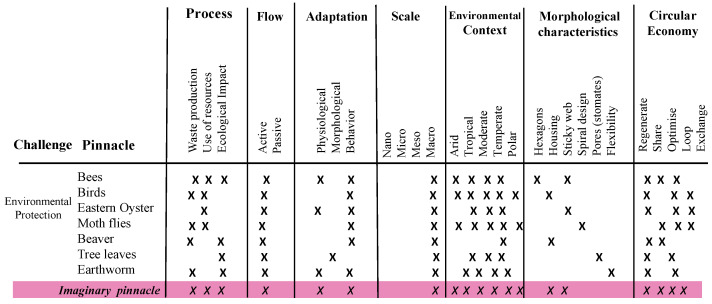
Pinnacle matrix analysis for the environmental protection challenge.

The environmental protection pinnacles analysis matrix is presented in Figure 6. The relevant features of this challenge are highlighted with the color pink. Their results show that the three defined processes coincided with pinnacles, with active flow and physiological and behavioral adaptations. The macroscale means the natural scale in which the solutions are carried and, since the application is for construction, all types of weather are applied in the environmental context. Within the morphological characteristics, there is that of housing and sticky network and, in circular economy, four of the five characteristics were applied—regenerate, share, optimize and cycle.

**Figure 7:**
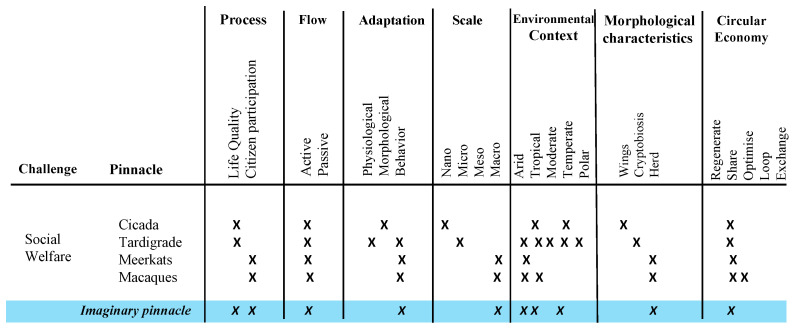
Pinnacle matrix analysis for the social welfare challenge.

The social welfare challenge’s pinnacle analysis matrix is presented in Figure 7, where the two processes were taken for the imaginary pinnacle and the active form and macro-scale dominated. In the environmental context, the arid, the tropical and the temperate climates were predominant. In morphological characteristics, the herd was emphasized and, in the circular economy, the characteristic of sharing was.

**Figure 8:**
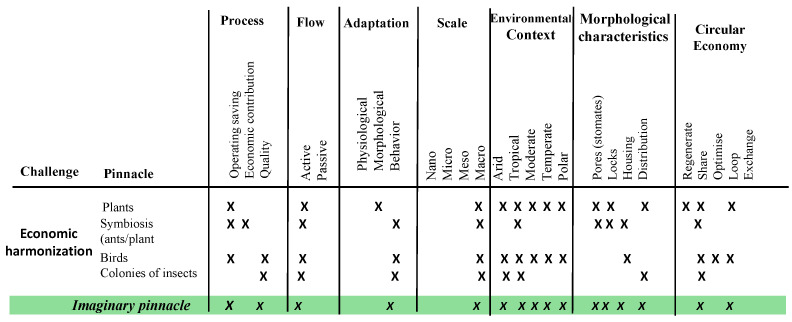
Pinnacles Matrix Analysis for the Economic harmonization Challenge.

The results for the pinnacle analysis matrix in the economic harmonization are presented in Figure 8. Operational savings processes predominated over quality. In adaptation, behavior was predominant and, in scale, macro was dominant. All morphological and environmental context characteristics coincided with the imaginary pinnacle, contrary to the circular economy, where only “sharing” applied.

**Figure 9:**
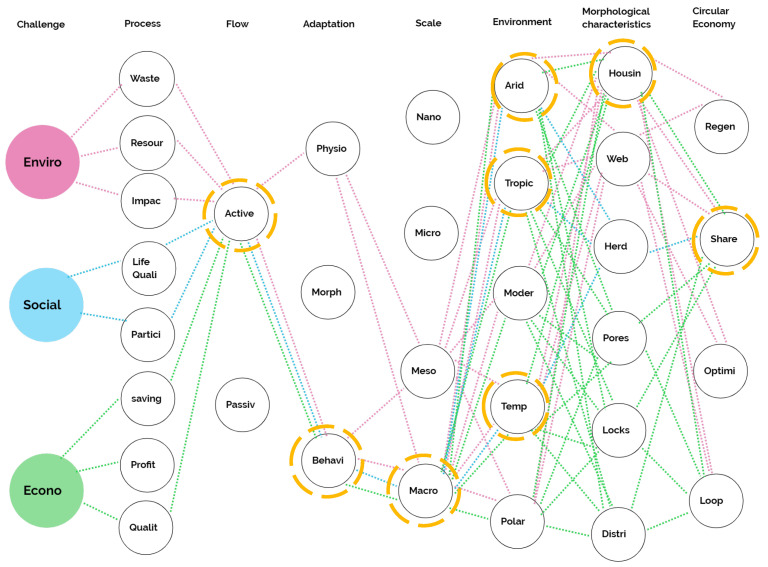
Design path matrix.

Based on the pinnacle analysis matrix, we established the specifications for the design path matrix, where each imaginary pinnacle was classified and categorized with its corresponding trajectory [40]. Each vertical column represents a category and its various characteristics. The pink, light blue and green dotted lines in Figure 9 denote the trajectory of the imaginary pinnacles. The orange nodes emphasize the dominant characteristic of each category, representing the design concept’s trajectory to address the problem and its approaches. These nodes contain the highest number of connections in the “challenge” trajectory, with the highest number of connections being the most dominant per category.

#### 3.1.2. Road Map Definition

**Figure 10:**
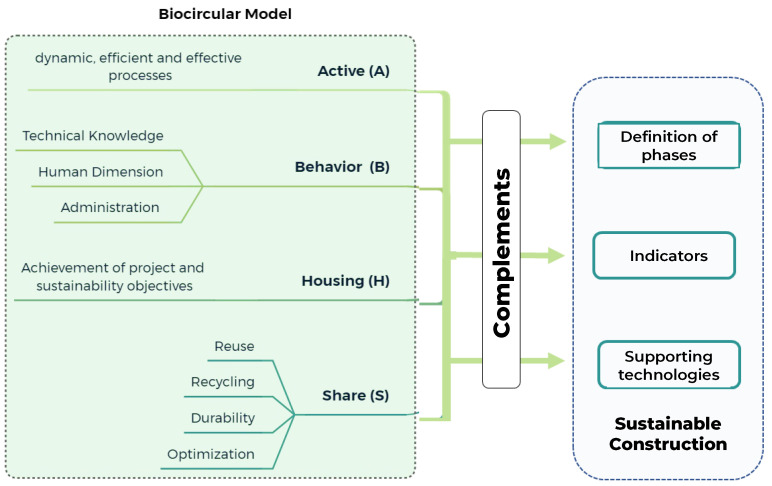
Biocircular model and its influence on sustainable construction.

The design pathway matrix established the dominant characteristics in each category, considering the challenges of sustainability and circular economy. The active flow was predominant in the face of environmental protection (1), social welfare (2) and economic harmonization (3), indicating that the processes that would lead to sustainable construction must be dynamic, efficient and effective to meet the challenges presented. Likewise, for the three challenges, the influence of behavior prevailed, giving significance to the management of administration, technical knowledge and skills for construction, with the macro scale being the natural one. For the environmental context, all the elements were reviewed, highlighting the arid, tropical and temperate climates, pointing out the safety considerations that would change according to the type of project, location and attributes. In the morphological characteristics, housing was dominant, where its interpretation would be the provision of facility or service for which it is built. Finally, for the inclusion of the circular economy, sharing stood out from the ReSOLVE framework [17]. This emphasizes asset sharing, reuse and durability through maintenance, design and retrofitting. The inclusion of these elements would complement the definition of phases of the sustainable construction process, the evaluation of indicators in the literature and the relationship with technologies to support the process (Figure 10).

(a) Supporting technologies

These tools enable better collaboration between stakeholders and multidisciplinary teams, reducing uncertainties and decreasing contingencies, risks and costs [32].

The new emerging technology of “Building Information Modeling (BIM)” has been promising for architecture, engineering and construction. This tool allows stakeholders to make decisions regarding sustainability in the early design and pre-construction phases by its concept of collaborative design of a universal computational model, which handles multidisciplinary information to be integrated into a model and motivates the analysis of environmental performance and sustainability metrics with a life cycle approach in these areas [32]:

- Construction orientation;
- Shape of construction;
- Natural lighting analysis;
- Water supply;
- Sustainable materials;
- Site and logistics management.

Geographic Information Systems provide opportunities for sustainability and cost saving in extending, operating and maintaining the built environment. The most widely used technologies in the industry are BIM and other traditional technologies, such as computer-aided design (CAD) systems for designing and storing building information. However, GIS complements BIM or CAD files in connecting relevant information at the site, municipal, or regional level [32] due to the following:

- It enables a more coordinated view and increases collaboration and understanding while reducing risks and associated costs.
- It provides visualization, analysis and comparison of possible alternatives to improve performance.
- It contains analytical tools necessary for stakeholders to decide which solution is the best to achieve in the short and long term.
- It supports the construction industry in the transition to sustainability, identifying green practices and patterns.

(b) Project Management for Sustainable Construction

To achieve the expected results of the project, three types of indispensable variables must be harmonized [36]:

- Technical dimension, namely, the areas of knowledge relevant to the nature of the project to be executed for its proper fulfillment, given a team of professionals to apply it.
- Human dimension, namely, aspects that can condition the success or failure of the project among all stakeholders. These include coordination, negotiation, participation, motivation and integration.
- Management, namely, where the work of the various resources is integrated and reconciled decisively for the production and fulfillment of results.

The success factors for any project, traditionally, include the final result, costs and time. Nevertheless, our route with the biomimetic and circular economy approach integrates sustainability challenges as another success factor in construction [13,36].

It is also essential to identify the stages of a project, since the motivation in this field of research is that not only the final products are green but so are the process [32] and what it entails, i.e., the identification of activities, deliverables and allocation of responsibilities among the executing team. In such a way, the abstraction of the dominant elements in the design path matrix is given, along with the definition of what each stage entails in sustainable construction. These descriptions are supported by the exploratory review of the stages described above in Figure 10.

### 3.2. Case Study Definition: Expert Assessment

A survey was distributed to construction professionals within the emulation phase to assess the level of acceptance of the phased definitions with the considerations of the biocircular model, by initially identifying the following at a personal level:

- Job position (director, designer, engineer, project manager, or contractor).
- The sector to which they belonged (public, private, or independent).
- Years of experience (less than 5 years, from 5 to 10 years, from 10 to 20 years, or more than 20 years).

Then, the first section, regarding consulting with professionals for knowledge in sustainable construction, biomimicry and circular economy, was presented for control. This included eight questions consulting the phases they identified for a construction project; the measurement by scale, from null to very high, of the knowledge of construction, biomimicry and circular economy; the most important attributes required to define sustainability in construction among those suggested; and the selection of the aspects considered the most important for the challenge to achieve sustainability in construction.

A second section regarded the proposed road map with the biocircular approach, in order to obtain evaluation and degree of acceptance of the proposal along with recommendations. A comparative table of the stage definitions was presented conventionally on the left and the right, with the biocircular approach highlighting the elements that sustainable construction contributes.

The third section was the SWOT analysis, presenting possible barriers found in [42] for selecting respondents, such as challenges, risks and difficulties of implementation, in the context of Panama. For opportunities and strengths, as well as perceived challenges, factors [43] were presented to be select the importance of which had to be established.

In addition, a final section for additional comments and recommendations was provided in a non-binding manner.

## 4. Results Analysis and Discussion

The following section presents the analysis of the results of the biocircular model implementation in each of the life cycle phases in sustainable construction projects, along with a brief discussion regarding the acceptance of such biocircular approach by experts in the field.

### 4.1. Application of the Biocircular Model to the Sustainable Construction Phases

As a complement, the biocircular model (Figure 10) approach was applied to the sustainable project phases’ definition. Table 2 shows how each approach of the biocircular model impacts and complements each phase of the sustainable construction project phases.

(a) Initiation

During this phase, the groundwork is laid for proper project management and for the establishment of a common understanding of the critical elements for all stakeholders (from owners to community representatives) [2,36]. These elements would be the goals, objectives, scope, site selection and budget, along with the consensus to integrate sustainability for specifications and practices [11,23]. Therefore, it implies hiring relevant human resources for planning, design and management, as well as for feasibility and risk studies [2,11].

The budget should emphasize life-cycle costs, shifting the focus from short-term return on investment to long-term gains in green and local materials, material reuse, eco-efficient design and operational savings [2,25,43].

It is important to highlight the role of stakeholders, those who can influence or be influenced by the project, the conditions established by these stakeholders in the objectives and the risks to be considered for its completion [25].

(b) Planification

This consists of the detailed preparation of the regular work plan of the project staff, recognition of collaboration between technical sectors and the definition of tasks and milestones for the design. More detailed management plans are developed in terms of resources, quality, time, budget and procurement. In the sustainable context, the environmental impact assessment study in the area of influence is included, where the direct and indirect environmental consequences of the project’s activities on the physical, biological and socioeconomic environments are recognized. This includes an environmental management plan that includes recommendations for mitigating impacts, preventing risks, providing environmental education and establishing mechanisms to include the public in decision making. Complementing the sustainable efforts, a waste management plan must be implemented, aligned with recovery and recycling. The main guidelines of the detailed project specifications, the green factors and the correlation with environmental policies that apply [11,23] are also constituted in this phase.

To achieve the defined, a solid understanding of the project specifications, factors and green benefits must be provided to the work team, since a critical green capability must be developed for the selection of sustainable materials, which is based on durability, costs, maintenance, local and recycled components— where sourcing locally would mean to reduce emissions from transportation and the promotion of reuse and recycling [11,18,23,43]. Social aspects include occupational health and safety programs for implementation and collaboration with suppliers to achieve environmental objectives [33].

Similarly, project progress and quality metrics should be defined, which could be qualitative or quantitative for the monitoring and control stage, to optimize the path of milestones and activities necessary to meet the scope.

(c) Design

This phase contains the use of best practices to maximize the design results of the construction project, the evaluation of the costs of sustainable strategies for environmental benefits and the accuracy of combinations of design strategies [23]. Among its categories for reducing environmental impact there were variations according to the type of construction project, but the following stood out: materials and resources, waste reduction, energy efficiency, water savings, comfort, site management, recycling and reuse—with a view of adaptive and environmentally friendly designs [7,23,24,32]. Design considering the most efficient use of natural light and ventilation, safety in case of environmental accidents and the legal requirements that apply are among the most prominent [7,11,32]. Thus, the integration of qualified contractors and subcontractors is essential to reach a common design vision and avoid future problems [11].

Considerations at this stage [18,25,43] are as follows:

- Increase in or maintenance of green space;
- Reuse, recycling or recovering of materials or parts of materials;
- Design for reduction in material, water and energy consumption;
- Avoidance of using materials that become hazardous waste;
- Consideration of the use of renewable energy;
- Adaptation of design options to environmentally impactful scenarios (waste production, emissions, etc.);
- Recovering of water and energy;
- Long-term planning for climate change risks and their effects;
- Modular design;
- Innovation capacity.

(d) Construction

This phase consists in the execution of construction and general mobilization work in an effective manner, with a focus on minimizing resources, waste and emissions to reduce ecological impacts [11,25,32]. It is important to contemplate periodic environmental education and training on the sustainable construction strategies that apply, including the specifications and technologies that would be used to optimize processes [2,11,43]. Compliance with the work schedule, quality management systems, safety and health in construction are other factors in this phase [24].

For construction, the environmental management plan containing the environmental management procedures must be carried out, particularly, the management of air and noise pollution, water and energy use, occupational health and safety, resource utilization, transportation and emissions, solid waste, quality standards and legislation [7,25,31]. For solid waste, waste quantity reduction and sorting for recycling or sale to recycling facilities need to be considered [11].

Some sustainable actions that can be carried out are [4,18] the following:

- Reuse of elements—building components, rubble, concrete, steel and wood;
- Having containers for waste sorting on site;
- Installation of efficient plumbing for water use;
- Purchase of nearby available material to reduce air pollution produced by vehicles;
- Reuse of excavation materials for backfill;
- Increase in or maintenance of green areas;
- Limitation of tree, soil and habitat disturbance.

(e) Monitoring and control

In the framework of project management, this is a decisive phase for the success of a project and its implementation is continuous from the beginning, since it allows stakeholders to verify whether the project plan is being implemented as planned or whether certain actions need to be corrected. It includes receipt and authorization of work, change and version management, analysis and performance reports on integrating sustainable factors and corrective and preventive actions [36]. Thus, meetings among project stakeholders, as well as inspections, should be performed with high frequency to identify progress over time, verify the performance of sustainable actions to minimize impacts (emissions, waste and material consumption), quality assurance, risk prevention and problems to be addressed on time [2,11,43].

(f) Delivery

This stage is where the project is finalized and ready to be handed over to the owner. A checklist and tests are performed to ensure compliance with criteria and the project’s environmental objectives to verify operational performance and quality. It is important to obtain the satisfaction of all stakeholders [2,11,23]. For the sustainable construction context, the lessons learned and the capacity of building with this process, its proper completion and the milestones it would bring in terms of reputation are all important [43].

Now, the continuation stages are no longer of the process but the product. In general, these stages are intended to provide a guide for operation and maintenance according to the type of project that applies. These plans should combine cleaning, work practices, training and surveillance [25]. Emphasis should also be placed on the constant monitoring of environmental and social performance metrics to be applied to environmental management, energy, carbon footprint reduction, solid waste, landscape, water and environmental education campaigns [24].

If abandonment is included, options for transformation and adaptive reuse should be evaluated, as well as the use of materials to recover energy and prioritize disassembly and transformation over complete demolition [18].

### 4.2. Application of the Biocircular Model to the Sustainable Construction Metrics

Complementing the road map with the biomimetic and circular economy approach, 12 articles with indicators were identified in the literature review for the construction life cycle. These were filtered from the general to the specific, giving 7 articles with proposed indicators for the evaluation of the biocircular model: 11 quantitative indicators and 19 qualitative indicators, presented in Table 3 and Table 4.

For these, only four qualitative indicators complied with the four concepts of the biocircular model, namely, reuse of construction elements (earth, concrete, steel, steel, wood and other components), reuse of excavation materials for backfill, use of local material to reduce emissions and water reuse system. These account for 21% of the total qualitative indicators. Within compliance of three concepts, five indicators were found, giving 47% among the qualitative indicators. For the quantitative indicators, no indicators were found that complied with the four concepts; however, 6 of the 11 (54%) complied with three concepts. For future studies, indicators that comply with the biocircular model can be defined.

### 4.3. Experts Assessment to the Proposed Biocircular Model

**Figure 11:**
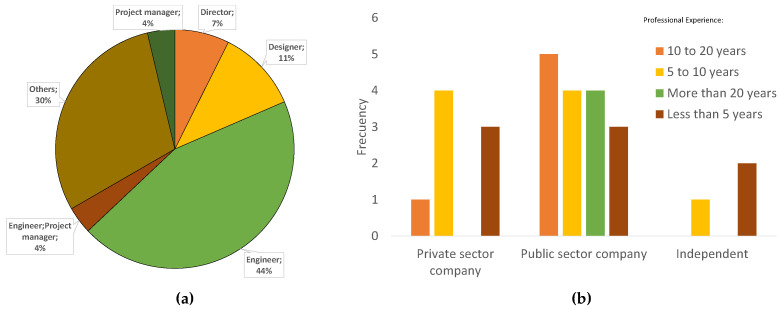
Professionals surveyed: (**a**) by job position and (**b**) by job sector according to years of experience.

Twenty-seven responses were received from professionals (Figure 11), with the most predominant among the defined options being the positions of engineer, with 12 respondents (44%), and designer, with three respondents (11%). The other option was the second most frequent, with 30%. The majority were from the public sector and were present in all the established experience ranges. The most frequentely selected option, among professionals, was 5–10 years of experience.

**Figure 12:**
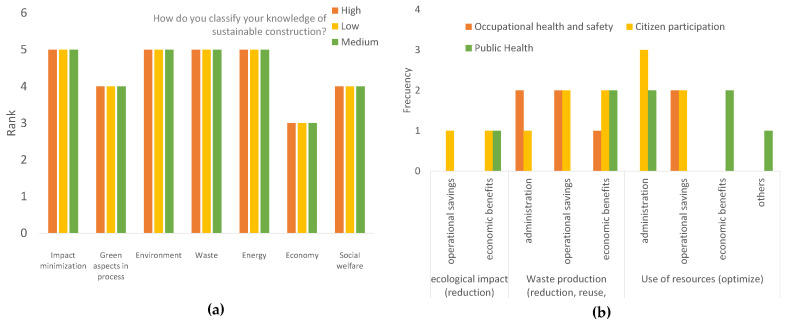
Results for: (**a**) attributes considered the most important for defining sustainability in construction and (**b**) the most important aspects considered to achieve sustainable construction.

The classification of attributes considered the most important to define sustainability in construction, presented in Figure 12a, was highly pronounced and coincided among professionals, regardless of their knowledge in sustainable construction (high, medium, or low). The most influential, for the definition, was the minimization of impacts, environment, waste and energy. The least considered for the definition was the economy. This reflects the need to join efforts to raise awareness of the benefits for investors of sustainable construction and its concepts of savings in materials, design and operation.

Dividing by sustainability challenges considered for the biomimetic methodology (Figure 12b), social welfare and citizen participation predominated, this being the fundamental social aspect for construction, since it is linked to the acceptance of projects in the influenced area. Economic harmonization was more closely contested among respondents, with economic benefits being the most popular by only one-tenth. This means that the aspects placed were considered of equal importance and influence. Finally, for environmental protection, the perception was equal for the factor of waste production (minimize, reuse and recycle) and resource utilization (optimize). These factors are important for reducing the ecological impact of construction. Additionally, they were asked if they would consider the inclusion of sustainability and unanimously agreed.

**Figure 13:**
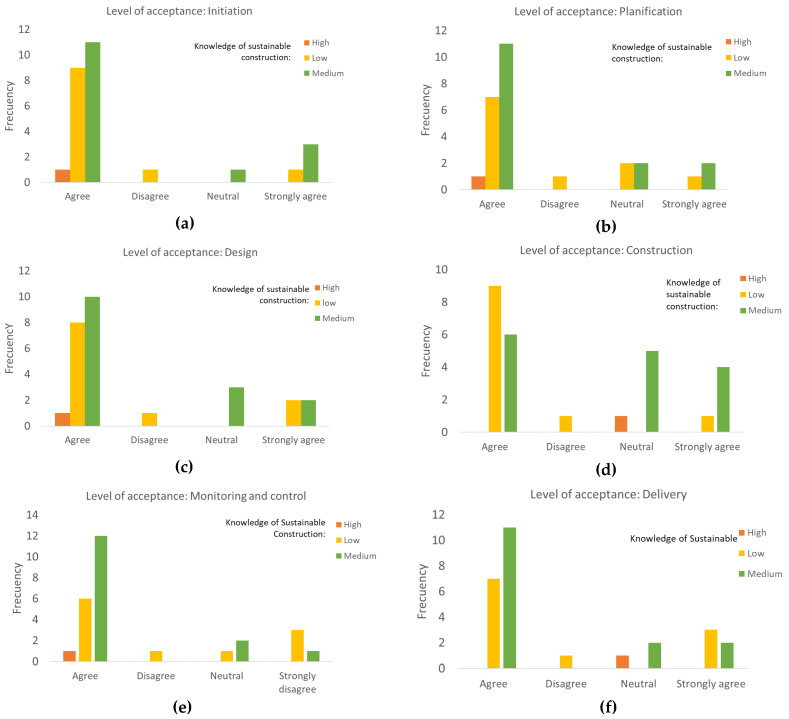
Results of phase acceptance based on the level of knowledge about sustainable construction: (**a**) initiation, (**b**) planning, (**c**) design, (**d**) construction, (**e**) monitoring and control and (**f**) delivery.

The levels of acceptance of the phases with the biocircular approach are presented in Figure 13. It should be noted that the definitions presented in the comparative table are a summary of the complete text for each one in this article.

As depicted in Figure 13, the phase with the best reception was the initiation phase (a), with 93% agreeing, including high, low and medium knowledge in sustainable construction. Only one professional stated that he disagreed and, by his own consideration, his knowledge in sustainable construction was low. This means that the importance of establishing early on the objectives and reaching a common vision among the stakeholders is of great interest for the approval of the professionals.

The phases of design (b), monitoring and control (e) and delivery (f) had 85% approval. One professional disagreed and four were neutral. There were variations in the distribution between agreeing and strongly agreeing. Delivery was the one with the highest frequency and the highest approval score, which could be due to the definition with the objectives and procedures. However, the participant with high knowledge weighted his perception as neutral.

For the planning phase (c), there was an acceptable 81% and, as it was identified among the patterns by the literature review, planning and design were binding; perhaps the summary presented should have shown more justification in the activities. Among the knowledge of sustainable construction, agreeing had a greater presence of levels, including high.

With 73%, the definition of the construction stage was the lowest among the six and, according to professionals with high and medium knowledge, a neutral position was maintained. Therefore, the activities and the sustainability factor in these activities should be studied in greater depth.

**Figure 14:**
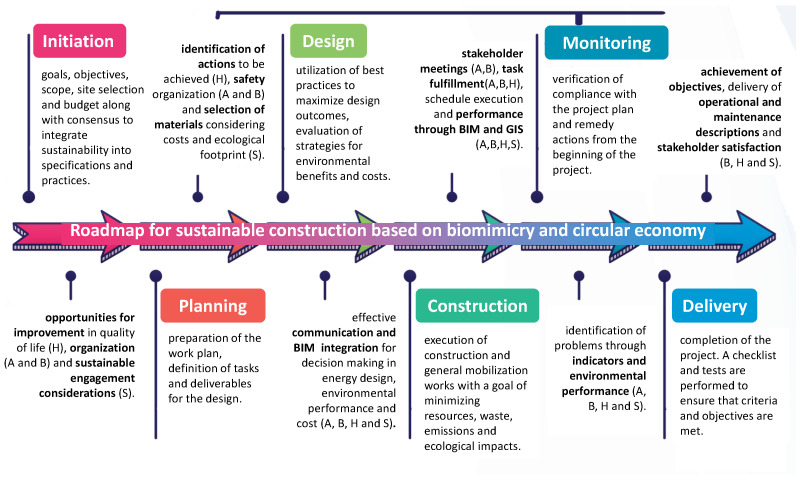
Summary of the road map phases definition with the accounting for the biocircular model approach.

In general terms, the definitions of this road map with a biocircular approach had a 83% acceptance among those consulted (Figure 14). Among the recommendations, there were the following:

- Give greater publicity to sustainable construction and circular economy issues, addressing designers, builders and investors;
- Consider design and material safety standards to be implemented;
- Present BIM opportunities in more depth (general aspects were included in the survey, but not the technology itself and its opportunities).

### 4.4. SWOT Analysis of Proposed Biocircular Model

The survey included a bank of suggestions for sustainable construction in terms of barriers and opportunities. In these, the professionals categorized them into strengths, opportunities, skills and threats, which are shown in Table 5. It was emphasized that the professionals identified these challenges in the context of Panama, in consideration of the lack of knowledge of sustainable construction practices, the lack of governmental support and human attitudes towards change. Similarly, the strength in terms of reputation, automation in strategies and waste optimization were highlighted and the opportunities presented in terms of economic benefits and training.

## 5. Conclusions

This study represents a valuable opportunity for the field of project management and sustainable construction, because it includes the application of the biomimetic methodology to unify its definitions and elements and it allows the circular economy to be innovatively and creatively included in the activities by emulating nature and its self-sustainability. In this way, a defined framework is obtained for all stages of construction, the considerations to be taken within their practices and evaluation of quantitative and qualitative indicators applicable in the phases. Likewise, the exposure of benefits for the environment, investors, designers and contractors through the optimization and automation of the process itself, the costs, risks and success are considered.

In the context of Panama, this study symbolizes a precedent in the field and sensitization of professionals with experience in the country on the relevance of the topic, concepts and components to influence the construction and transform it towards sustainability. In line with this, relevant recommendations can be stated as follows:

- Give relevance to each stage of the sustainable construction process, mainly the earliest ones and those of follow-up and control, to take corrective measures and evaluate performances.
- Invest in green technologies for waste management, water and energy savings, as well as their sources.
- Train personnel in sustainable construction, health and safety practices and sensitize them with ongoing environmental education, e.g., as in [44,45].
- Maintain collaboration among all project stakeholders and ensure that they master the benefits of sustainability and circular economy.

Finally, future work could validate the biocircular model within a case study of construction and make improvements, as well as contemplate specific practices within the phase definitions. Among the limitations, we report the summary of the context of the research and the definitions of each phase for the survey distributed to professionals with low background in the subject and its applicability to the different types of existing construction.

## Figures and Tables

**Table 1:** Elements for defining sustainability in construction.

Elements	Occurrence * (%)	References
Impact minimization	75	[2,4,7,8,11,18,20,23,24,25,26,27,28,29,30]
Resources efficiency	50	[2,4,8,18,20,26,28,29,31,32]
Green aspects in each phase	75	[7,8,11,18,20,23,24,25,26,28,30,31,33]
Environment	75	[2,7,8,11,26,27,28,29,32,33,34,35]
Economy	75	[4,7,8,11,25,26,27,28,32,33,34,35]
Health	25	[7,11,25,27,28]
Energy	50	[7,11,20,25,26,27,32]
Safety	25	[7,11,27]
Social aspect	75	[4,7,8,25,26,27,28,32,33,34,35]
Waste	50	[7,8,18,25,26,29,30]
Triple Bottom Line	50	[4,7,8,26,27,28,32,33,34,35]

**Table 2:** Biocircular Model Approach to each Sustainable Construction Phase.

Phases	Biocircular Model Approach			
	Active (A)	Behavior (B)	Housing (H)	Share (S)
**Initiation**	Organization among stakeholders on critical elements and productive capacity at the stage		Economic and social benefits of the project (opportunities to improve quality of life)	Considerations and scope
**Planification**	Organization in construction and occupational safety in terms of quality, control and maintenance		Identification of actions to be achieved	Selection of materials for the circular economy considering costs and ecological footprint
	-	Planning of the work team in the areas of knowledge with responsibilities		
**Design**	Integration of BIM as a decision-making tool, allowing the following to be performed: for energy design guidelines, environmental performance assessment, cost estimation according to the variety of design options, etc.			
	Effective communications among designers, clients, environmental specialists and government to ensure that all requirements are incorporated		-	-
**Construction**	Stakeholder meetings before project milestones are initiated or completed		-	-
	Compliance with occupational health and safety measures		-	-
	Execution of the schedule of activities on time and compliance with quality standards			-
	Collaborative emission monitoring among all members (including subcontractors) on-site through BIM and optimization of rolling equipment routes to decrease emissions			
**Monitoring** **and** **control**	Identification of issues through performance indicators and BIM, as well as management responsiveness.			Performance monitoring of measures related to the reduction oin emissions, solid waste, wastewater, material consumption and environmental risks.
**Delivery**	-	Compliance with environmental objectives, correct operability, delivery of maintenance descriptions and stakeholder satisfaction.		

**Table 3:** Quantitative Indicators evaluated by the Biocircular Model.

Reference	N°	Quantitative Indicators	Phases						Biocircular Model			
			I	P	D	C	M	De	A	B	H	S
[8]	1	Job creation (N°)	*			*			*	*		
	2	Rate of return (cost-benefit) ($)	*	*						*	*	
	3	Net income ($)	*	*					*	*	*	
	4	Complaints (N°)				*	*	*	*	*		
[28]	5	Training of staff in environmental awareness (N°)		*	*	*			*	*	*	
[29]	6	Monitoring and compliance inspections (N°)				*	*		*	*	*	
	7	Equipment maintenance (N°)		*		*	*		*	*		*
[34]	8	Amount of water saved (m${}^{3}$)		*	*	*				*	*	
	9	Amount of water recycled (m${}^{3}$)		*	*	*				*	*	*
	10	Amount of energy savings (kWh)		*	*					*	*	
[42]	11	Follow-up of the Environmental Management Plan, ratio of objectives reached		*	*	*			*	*	*	

**Table 4:** Qualitative indicators evaluated by the biocircular model.

Reference	N°	Qualitative Indicators (Yes/No)	Phases						Biocircular Model			
			I	P	D	C	M	De	A	B	H	S
[4]	1	On-site waste separation		*		*			*		*	*
	2	Reuse of construction elements (earth, concrete, steel, wood and other components)		*	*	*			*	*	*	*
	3	Efficient plumbing systems for water use on construction sites		*		*			*		*	*
	4	Reuse of excavation materials for backfill		*	*	*			*	*	*	*
	5	Use of local material to reduce emissions		*	*	*			*	*	*	*
[8]	6	Habitat changes				*		*	*	*		
[28]	7	Use of raw materials with recyclable content		*	*	*			*		*	*
	8	Installation of energy saving lamps		*	*					*	*	*
[29]	9	Coverage for air pollution reduction		*		*					*	
	10	Water reuse system		*		*	*		*	*	*	*
[32]	11	Risk safety considerations		*	*	*	*		*		*	
[34]	12	Use of clean energy		*	*						*	
	13	Improvements in area services		*	*						*	*
	14	Citizen participation	*	*			*		*	*	*	
	15	Inclusive facilities		*	*	*				*		*
[42]	16	Stakeholder participation (requirements and interests)	*	*	*	*	*	*		*	*	
	17	Organizational culture		*	*	*	*		*	*		
	18	Social responsibility	*	*	*	*	*		*	*		
	19	Transparency in processes and policies		*	*	*			*	*		

**Table 5:** SWOT analysis obtained from the survey.

Strengths		Opportunities	
1. Automation in design estimation, costs and strategies (BIM)		1. Focus on reduction in material and energy consumption	
2. Motivation to apply green technologies and methodologies		2. Waste reuse, recovery and recycling	
3. Optimization of processes reducing environmental impacts		3. Automation in design estimation, costs and strategies (BIM)	
4. Good reputation		4. Cooperation with staff and suppliers to meet sustainable goals	
5. GHG emissions monitoring (GIS and BIM)		5. Motivation to apply green technologies and methodologies	
		6. Choosing quality and environmental design certifications	
		7. Economic benefits from eco-efficient materials	
		8. Training of personnel in environmental issues	
		9. Reduction in contamination in physical media (air, soil and water)	
		10. Waste reduction	
		11. Reduce frequency of environmental accidents	
		12. Improve the company’s operational capacity	
		13. Improving personnel skills	
		14. Socio-environmental responsibility	
		15. GHG emissions monitoring (GIS and BIM)	
**Risks/Threats**	**Rank**	**Challenges**	**Rank**
1. Variation in material prices.	3	1. Lack of knowledge of sustainable construction practices.	5
2. Lack of technical knowledge.	4	2. Lack of environment-friendly materials.	4
3. Delay in decision making.	3	3. Lack of accessible guidance.	4
4. The price of the internship application.	4	4. Resistance to change in the adoption of new practices.	3
5. Lack of customer demand.	4	5. The application price of sustainable practices.	4
6. The fragmented nature of the industry.	4	6. The customer is concerned about profitability.	4
7. Poor management and communication.	4	7. Lack of knowledge of the benefits.	4
		8. Time for implementation of new practices.	4
		9. Lack of government support.	5
		10. Human attitudes to change.	5
		11. Poor management and communication.	4

## Data Availability

Not applicable.
